# Notes and Comments

**Published:** 1924-06

**Authors:** 


### NOTES AND COMMENTS.

### GRANDIOSE BUT IMPRACTICABLE.

IT is already clear that the Government's Housing Bill is too ambitious and too costly to produce the results expected by its authors. It is even becoming doubtful whether it can be got to work at all. It is based on the extravagant assumption that the country will require two and a-half million houses in the next fifteen years. But we do not need accommodation for anything approaching twelve or thirteen millions of people in a decade and a-half —the population, happily, does not increase in that ratio. There is to be a subsidy of £13 10s. for every house built under the Bill, £9 of which would come out of the Treasury, the remainder being found by the local authorities. This means a capital cost of 603 millions spread over a period of forty years, and at the end of the fifteen years an annual charge of thirty-seven and a-half millions. The capital value of the subsidy is more than double that of the £6 per house for twenty years provided for by the Chamberlain Act, yet the builder who commutes will not be allowed to take more than he could under that Act. Apart, however, from the outstanding difficulties of finance on this colossal scale, the Government are faced by even more immediate obstacles. The prices of building materials are rapidly rising, and are already substantially higher than they were when Mr. Wheatley arranged that, in return for a certain measure of dilution, the building trades should be guaranteed work for fifteen years. But what is the value of such a guarantee when the local authorities reserve to themselves the right to delay or abandon building at any moment and for any reason whatsoever ? In the anxiety to do things on a grandiose scale the grave mistake has been made of not allowing the Chamberlain Act to go quietly on its way. It was fulfilling its object by producing houses at a rapid rate, and it is very doubtful whether Mr. Wheatley's scheme would produce them as rapidly, since it carefully endeavours to fend off private enterprise. It is, in fact, possible that it will never come into operation in its present form.

### THE BRITISH HOSPITALS ASSOCIATION.

THE fact that the annual Conference of the British Hospitals Association is this year to be held in London invests that important fixture with even more than its customary interest. It is appropriate enough that the Conference should be welcomed by Sir Humphry Rolleston as President of the Royal College of Physicians, and that, on the second day, the Council should be entertained to luncheon by the City Companies, whose hospitality is gracious and legendary. The City of London has always been foremost in hospital good works, and it is not for nothing that its bounds contain the Empire's most famous hospital. By an exercise of true wisdom the programme of the Conference, which will sit on Thursday and Friday, June 19th and 20th, has not been overloaded. Dr. F. N. Kay Menzies, Sir Napier Burnett's successor at the Red Cross, will read a paper on " The Relation of the Voluntary Hospitals to the Municipal Authorities," a subject which is certain to grow rapidly in importance. Sir Wilmot Hetherington will speak on " The Training of Nurses," and Mr. R. J. Meller on " Contributions to Voluntary Hospitals by Approved Societies," a subject at once momentous and difficult. Upon each of these topics there is room for lively and practical discussion, but, happily for the delegates, they will not be talking all the time, for on the afternoon of the second day they will have opportunities of visiting all the great teaching hospitals and most of the special ones.

### CERTIFICATION AND CRIMINAL RESPONSIBILITY

TWO events of peculiar interest relating to the law of lunacy have occurred during the month. The appeal of Dr. Adam and Dr. Bond from the verdict and the heavy damages against them in the remarkable Harnett case has succeeded. The Court of Appeal has, however, ordered a new trial in the case of Dr. Bond. We need not enter into the legal and technical reasons underlying the judgment of the Court of Appeal, the more especially since Dr. Bond's case is once more *sub judice.* It is well, however, to point out that nothing has happened to relieve the public mind about the defects of the law. As that law stands certification is too easy, and the rights of the patient are too little recognised. He is not formally and officially informed of the reasons for his confinement, nor is a copy of the certificate served upon him ; he is in a far worse position than a prisoner who is being tried for an offence. The criminal side of the question has been raised in Parliament by Lord Darling's Criminal Responsibility (Trials) Bill, which sought to widen the existing law by providing that an accused person is entitled to a verdict of " not guilty " on the ground that he was insane " if at the time the act was done or omission made he was suffering from such a state of mental disease as therefrom to be wholly incapable of resisting an impulse to do the act or make the omission." This, broadly speaking, is the doctors' view. The lawyers' view is sufficiently indicated by the fact that of twelve judges consulted by Lord Hewart, ten were emphatically opposed to the Bill, one was doubtful, and only one was in its favour. It is therefore not surprising that Lord Darling's measure was rejected by the Lords on the Second Reading. Moreover, the inherent difficulty of the whole subject is increased by the fact that even people who can distinguish between right and wrong may be, and sometimes are, subject to " uncontrollable impulse."

### HOSPITAL SUNDAY.

FINANCIAL and political conditions, directly traceable to the long period of war, have of late reacted unhappily upon Hospital Sunday, and both in 1922 and 1923 the occasion produced less than had been expected. This year the day falls upon June 22nd, and it is especially desirable that the result should be an advance not only upon those but upon all previous years. The needs of the London hospitals are larger and more urgent than ever, and the pressure upon their resources increases, not steadily but very rapidly, and final emergence from the difficulties which have beset them for the last ten years will be possible only by a substantial and continuous increase in the means at their disposal. Success is more important than ever in view of the renewed demand that the hospitals should be placed in the care of the State and run at its cost. A " record " collection would help to make it clear that the public are determined to maintain that voluntary system which has hitherto been the glory of the hospitals of this country, and to maintain it because they are satisfied that it is the most efficient and the most dignified system. In little more than half a century Hospital Sunday has produced close upon three millions of money, and it is easy to realise the difficulties the hospitals would have had to face without this help. The Fund now provides 10 per cent. of the charitable income of 250 hospitals and similar institutions, to say nothing of what may be called its private benevolences. Such a Fund makes an irresistible appeal to the head as well as to the heart, and we hope that on Sunday, June 22nd, it will be listened to more effectually than ever before.

---

### THE COMMISSION ON HEALTH INSURANCE.

IT is officially announced that the Terms of Reference to the Royal Commission are to be :— " To inquire into the scheme of National Health Insurance established by the National Health Insurance Acts, 1911–1922, and to report what, if any, alterations, extensions or developments should be made in regard to the scope of that scheme and the administrative, financial and medical arrangements set up under it." It will be seen that, as applied to the Health Insurance scheme, the reference is very wide, but that it does not extend to the subject of Unemployment Insurance or to any of the other matters which would legitimately come within an all-in scheme of social insurance which is being pressed in so many quarters. The Royal Commission will doubtless have submitted to it a mass of evidence from all the interested quarters. The difficulty will be to get evidence from disinterested quarters. There will be a considerable danger of the Commission becoming absorbed in intricate detail, and much will depend, in this as in other respects, on the chairmanship. Many general broad questions await the answer of the Commission. For instance, is the administration of the cash benefits by the Approved Societies the best that can be devised ? Has the underlying idea—the control of the insurance service by the insured themselves—come about in practice ? Has the separation of the administration of cash and medical benefits justified itself ? What is the justification in a national scheme, financed by uniform contributions, for different scales of benefits ? Should the medical services be made more comprehensive ? Should they be extended to the dependents of the insured ? What have been the effective results of setting up new bodies in local administration, the Insurance Committees for purposes of medical benefit, and of the experiment of giving the doctors a share in the administration ? Should the medical benefit become a local health service, and under what authority ? These and doubtless many other questions of real substance will afford the members of the Royal Commission a wide and intriguing field of study for a not inconsiderable period.

---

### HOW TO ARREST A HICCOUGH.

THERE are many domestic remedies for the relief of hiccough, such as sipping water in the most uncomfortable posture the patient can possibly adopt, or sucking a lump of sugar. Another device, as simple and more effective in children, is to test the hearing of each ear with a watch. As the child's attention is concentrated on detecting the tick of the receding watch, the hiccough ceases from the mere force of neglect. Unfortunately, the appearance of epidemic encephalitis, or sleepy sickness, has added greatly to the terrors of hiccough, which, as a manifestation of this disease, is a most distressing complaint, robbing the patient of sleep for many consecutive nights. Since it is due to clonic contractions of the diaphragm, the most rational treatment of it in a severe form would seem to be stretching this muscle by some means or other. In this connection Professor T. Hishikawa, of Japan, has lately described two devices by which it is possible to employ simple reflex mechanisms to cut short or block the impulses travelling to the diaphragm and giving rise to hiccough. The first device is to tickle the inside of the nose with a feather or strip of paper. The patient responds by sneezing, and in so doing he throws his diaphragm into violent action, and thus interferes with the rhythmic contractions giving rise to hiccough. The second device is equally simple but more disagreeable. A finger or some other object is introduced into the patient's throat, starting the vomiting reflex, which again induces violent contractions of the diaphragm, diverting it from its morbid avocation of hiccoughing. In the most severe cases of hiccough success has sometimes been achieved by passing a stomach tube and leaving its lower end in the stomach for a little while. But so heroic a remedy should be a last resort, and we are indebted to Professor Hishikawa for drawing our attention to measures which are at once effective and easy.

---

### THE QUEST OF THE COMPLEXION.

AMONG the young women of England there is at this moment no more unpopular man than Sir Arbuthnot Lane. He has actually dared to tell them that the real way to preserve their " school-girl complexions " is to take brimstone and treacle. That a man, even when he is a doctor, should venture to have views upon such a subject is just one proof the more of the steady encroachments of the male creature upon feminine preserves. And when those views take the direction of brimstone and treacle the outrage is flagrant. Numbers of indignant maidens who know that there is such a thing as treacle (in the nursery they rather liked it) and have heard of brimstone at all events in a theological connection, have been constrained to seek, often from their grandparents, information as to why the two should be combined. The answer has left them more profoundly convinced than ever that Sir William is a " stuffy old doctor." Every girl knows that complexions need external, not internal treatment. You rub a perfumed and expensive cream upon them, powder them delicately, and never wash them more than once a day. This is easy, simple and interesting, especially when a trifle of colouring matter is artistically added. How different the clumsy method of Sir Arbuthnot Lane! Many people like treacle, but brimstone is not exactly alluring, especially when your teeth come into contact with a thick brown lump. Yet the mixture is healthy and purifying, and very cheap. Our own complexion recipe would be cold water thrice daily, and brisk friction with a fleecy towel. We do not pretend that there is anything artistic about this, and we are painfully aware that it suffers from the fatal objection of being the natural treatment of the human skin.

### HEALTH BY WIRELESS.

THERE is plenty of evidence, says the Surgeon-General of the United States Public Health Service, in his recently published report to Congress, that health information sent out by radio is having a definite effect. School teachers have copied these broadcasts in shorthand and used them in school. Local health organisations have installed receiving sets, and the service has brought popular education in health matters into remote regions, and touched a group of people hitherto uninformed in these subjects. There are, doubtless, great possibilities in this means of educating the uninformed public, but it must be done with discretion. Nothing could be worse for the cause of health progress than an unwise saturation of the citizen, who may soon display a desire to "go dry" in this connection. The report, which is for the fiscal year ended June 30, 1923, contains a mass of information of interest to observers and students in public health. It is interesting to note that in consequence of the enforcement of international agreements no cases of the major quarantinable diseases gained access to the United States during the year; that the plague work of the service has now practically "faded out of the picture"; and that the fundamental need throughout the rural districts is for the establishment of efficient local whole-time services.

### AN ECHO OF 1871.

IT is announced that the Scarborough Corporation have bought for £6,000 Londesborough Lodge, in that town, which until comparatively recently was one of the residences of the Lords Londesborough. Their intention is there to house the famous collection of sporting trophies bequeathed to Scarborough by the big game hunter, the late Colonel Harrison, of Brandesburton Hall. Time was when the British Empire felt a poignant interest in Londesborough Lodge, for it was there, in November, 1871, that the Prince of Wales, afterwards Edward VII., contracted the typhoid fever from which he nearly died. Why everyone who lived in, or stayed at, Londesborough Lodge did not die of typhoid was almost a miracle. Subsequent investigation showed that it was saturated with sewer gas. It was badly built and badly ventilated, and there were three communications with cesspools, two of which were actually under the house. The tides exerted such backward pressure on the sewers that the traps could not resist it and the house was thus frequently inundated with sewer gas. The Lodge stood at the summit of a long length of sewer with no openings for the escape of gas, and the drains were so placed that none of them could be inspected. Adjoining the Prince of Wales's room was a *cabinet* immediately connected with the cesspool underneath. His valet slept in a room six feet four inches high and three maidservants in a room only six inches higher; the offices were mainly below the ground-level. Many of the house party were ill in various ways; Lord Chesterfield died, and two of the servants also had typhoid. So much for Scarborough. Sandringham also was found to be unsatisfactory. The waters of the adjoining village of West Newton were, the *Lancet* found, "so highly charged with sewage that they do not rank with drinking water at all—they are like the effluent from a sewage farm." It is not surprising that one of the Sandringham grooms should have died of typhoid. These revelations caused a tremendous outcry, and it was asked if such things could exist in king's houses what was likely to be the state of things in less exalted dwellings? In view of recent denunciations of trapped pipes it is interesting to recall that as far back as 1871 the *British Medical Journal* described traps as "at the best very delusive contrivances." Yet we still continue to trap!

### THE CARE OF THE CONVALESCENT.

THE question of utilising fully the provision for the care of convalescents as a complement to hospital treatment has not received the attention which it deserves. An interesting paper has just been printed by the Public Health Committee of the New York Academy of Medicine embodying the results of a recent inquiry into this subject as affecting New York City, and it is found that the institutions with facilities for convalescents are utilised only up to 72 per cent. of their capacity. No more than seven of the sixty-two convalescent institutions are operated by hospitals. In general there is, in the arrangements of the convalescent homes, a complete absence of standards based on an accurate knowledge of therapeutic results, whether as applied to food, recreation, rest, or work. On the part of the hospitals themselves there is a lack of a generally accepted standard of selection of patients for convalescent treatment. As a result of the inquiry it is concluded that co-ordination of all the existing activities is badly needed as well as the formulation of adequate administrative and medical standards. A central registry or bureau of information could be made very serviceable in promoting these ends. It is a problem having most important bearings on the question of pressure on hospital beds needed in the heart of the city for cases of acute illness, which is by no means peculiar to New York City.

### SUN BATHS AND SHAVING.

WE are all becoming converts to sun-bathing, and are at last learning how powerfully curative a force the sun may be. Unluckily, it is only occasionally that we can adopt a costume meet for this kind of bathing. Moreover, in a land where the sun is seen with such comparative rarity, it is not always easy to pursue the "cure." Sir Herbert Barker, greatly daring, is, he tells us, invariably naked when he shaves. He is, we gather, impervious to weather, since he is as warm without his clothes as with them. He is immune from colds, sun and air having pigmented him. There is, of course, nothing new in the cult of early morning nudity, but it needs to be adopted with discretion. It would be more heroic than wise to begin naked shaving in December, and at any season something might depend on the behaviour of the razor. Back hair is understood to be a more serious matter than shaving—is that, also, to be conducted on Sir Herbert Barker's lines? He would like to see special places on the coast set apart for curative bathing in thin muslin "shorts" which permit of the penetration of sun-rays. Since, however, we are dealing with sun rather than water, this kind of bathing surely need not be confined to the coast. The open-air swimming-bath, which is growing in favour, should provide admirable opportunities, and in such places there ought to be little difficulty, given the presence of the sun, in basking in his rays in the "altogether." Let us not forget that, if only we give him the chance, the sun will prevent as well as cure.

### THE SCHOOL DOCTOR.

THE rapid quickening of the public conscience in regard to the welfare of the growing child finds remarkable expression in the development of the School Medical Services. The Annual Report of the City of Birmingham School Medical Officer, Dr. George A. Auden, for the year 1923 lies before us, and we have no hesitation in saying that those who do not keep in close touch with this question will find it a truly astonishing document. In it is to be found a record of the careful examination of 77,879 children, of whom 42,287 were discovered to have defects requiring observation or treatment. The staff available to the Education Committee includes the services of nine assistant school Medical Officers, thirteen dental surgeons, and also of ophthalmic, orthopædic and aural surgeons, anæsthetists, and a radiographer. A valuable feature of the Report is the full and interesting account given of an experiment on the nutritional value of an extra milk ration carried out in the case of thirty selected children, half of whom were poorly nourished. As a practical exposition of the value of the milk ration to the dietary of debilitated children the experiment proved highly successful, though Dr. Auden properly points out that a more extended experiment would be necessary before results could be tabulated which would satisfy statistical laws. Of the School Medical Service as a whole and its relationship with other ameliorative agencies, the medical officer observes that it can be envisaged from a few examples better than from statements of the mere volume of the work. He gives some carefully recorded histories of individual children, including the case of a delicate and undersized boy of seven, backward and listless, kept under constant medical supervision, who has now become the head boy of his school and captain of the school cricket team.

### PENSIONS FOR NURSES.

IT is notoriously difficult for the professional or working woman to save any substantial sum to support herself in old age. Women's salaries are still comparatively slender, and it is no wonder if the few pounds which can with the greatest economy be annually put by are often regarded as too negligible to save. This is particularly so in the case of the nurse, who rarely possesses any private means, and is even in these days paid less than the average skilled professional woman, such as the teacher or the secretary. Nurses, too, are extraordinarily generous—they will give their last farthing to help a fellow-nurse, but they are not as a rule thrifty. They need an inducement to save and some form of assistance in saving. A correspondent, writing to the *Times*, suggests that the Government should establish on a legal basis a suitable pension scheme for all nurses "who have faithfully and continuously served public bodies, hospitals and other institutions, and also give assistance to the private nurse in some fair and reasonable way." He points out that the work of nurses often demands a personal self-sacrificing service that cannot be directly paid for, and it remains a debt—a debt which at present is not sufficiently recognised. The number of nurses who, prematurely worn out, end their days in real poverty is unhappily large, and if some pecuniary help, in the form of a pension scheme on a self-contributory basis with State aid, could be afforded them, it would confer an inestimable blessing on a number of devoted women.

### THE CASE OF THE CHILD OFFENDER.

THE case of the child offender has always been one of peculiar difficulty, and Dr. Macnamara was on strong ground when, in introducing to the Home Secretary a deputation from the Voluntary Advisory Committee of the Children's Courts of the Medical Council of the People's League of Health, he urged that accurate knowledge of the mentality of delinquent children was essential if they were to be treated justly and satisfactorily. Hitherto the treatment of young offenders has too often been neither just nor satisfactory and the punitive aspect has been unduly prominent. This is the result, not of intentional harshness, but of a fundamental ignorance of child psychology, combined with a conviction that severity is necessarily the quickest road to reformation. It is this mistaken theory that has turned many first offenders into habitual criminals, and it is much to be hoped that the suggestion that the services of an expert medical man should be available in every Children's Court and that magistrates should be instructed to make use of this aid will be adopted. It should then follow that adequate means not for punishment, but for treatment would be forthcoming when needed. It would appear that the Home Office are decidedly in favour of some such system of observation, and Mr. Henderson read to the deputation an extract from a letter sent by his direction to an important local authority emphasizing the need of appointing for this purpose medical men to be attached to places of detention for juveniles.

